# A Novel Urease Inhibitor of Ruminal Microbiota Screened through Molecular Docking

**DOI:** 10.3390/ijms21176006

**Published:** 2020-08-20

**Authors:** Zhenyu Zhang, Ming Li, Xiaoyin Zhang, Nan Zheng, Shengguo Zhao, Jiaqi Wang

**Affiliations:** 1 State Key Laboratory of Animal Nutrition, Institute of Animal Sciences, Chinese Academy of Agricultural Sciences, Beijing 100000, China; zzy123779@126.com (Z.Z.); liming01@caas.cn (M.L.); zhangxiaoyin0426@163.com (X.Z.); zhengnan@caas.cn (N.Z.); 2 College of Animal Science and Technology, Huazhong Agricultural University, Wuhan 430000, China

**Keywords:** molecular docking, urease, inhibitor, rumen, microbiota

## Abstract

Inhibition of the urease activity of ruminal microbiota is not only beneficial for increasing dietary and endogenic urea-N utilization efficiency in ruminants but also might be applicable for the preservation of nitrogen fertilizer in soil and treatment of gastrointestinal and urinary tract infections caused by ureolytic bacteria. To discover urease inhibitors to efficiently target ruminal microbiota, the identified ruminal microbial metagenomic urease gene was used to construct a homology model to virtually screen urease inhibitors from the ChemDiv database by molecular docking. The GMQE and QMEAN values of the homology model were 0.85 and −0.37, respectively, indicating a good model quality. The inhibition effect of the screened urease inhibitor for ruminal urea degradation was assessed by ruminal microbial fermentation in vitro. The toxic effect of the candidate inhibitor was performed using gut Caco-2 cells in vitro. The results showed that compound 3-[1-[(aminocarbonyl)amino]-5-(4-methoxyphenyl)-1H-pyrrol-2-yl] propanoic acid (ChemDiv_ID: 6238-0047, IC_50_ = 65.86 μM) was found to be the most effective urease inhibitor among the candidate compounds. Compound 6238-0047 significantly lowered the amount of urea degradation and ammonia production in ruminal microbial fermentation. The 24 h degradation rate of compound 6238-0047 in ruminal microbial fermentation was 3.32%–16.00%. In addition, compound 6238-0047 (10–100 μM) had no significant adverse effect on the cell viability of Caco-2 cells. Molecular docking showed that compound 6238-0047 could interact with Asp359 in the active site and Cys318 in the flap region by the hydrogen bond and Pi-Alkyl interaction, respectively. Compound 6238-0047 could be used as a novel inhibitor for decreasing the urease activity of ruminal microbiota.

## 1. Introduction

Urease (urea amidohydrolases, EC 3.5.1.5) is a metalloenzyme, which carries two nickel ions in its active site [1]. Urease catalyzes the hydrolysis of urea into ammonia and carbohydrate [2]. Urease is well distributed among different bacteria, which enables them to survive in various ecological niches including the human stomach, soil, and the rumen of ruminants [3]. Excessive urease activity of urease producing bacteria makes them of great concern both in medicine and agriculture fields. For ruminants, ruminal microbiota take both exogenously and endogenously derived urea as nitrogen (N) sources for microbial protein production, which is an ideal protein source for ruminant production [4]. However, the excessive rate of urea hydrolysis in rumen makes it difficult for the efficient assimilation of ammonia with carbon skeletons to form microbial protein, leading to a waste of N nutrition for the animal and environmental N contamination. A urease inhibitor could efficiently decrease the ureolytic activity of urease and so it was considered to be a viable method for increasing the utilization of both dietary urea and endogenous urea for ruminal microbiota and ruminant animals, which holds great significance for animal production and environment N conservation [5].

Despite the significance of urease inhibitors, there is currently only one FDA approved urease inhibitor, acetohydroxamic acid (AHA), for human or animal use. Although the effectiveness of AHA in the treatment of urease activity related diseases such as kidney stone growth is proven, it is associated with significant adverse effects such as deep-vein phlebothrombosis and lower-extremity phlebitis [6]. In trials conducted on human patients with urease producing bacteria related diseases, more than 20% of patients suffered dermatological, neurological or hematological issues that have necessitated discontinuation of therapy [7]. In trials conducted on ruminal microbiota of ruminant animals, acetohydroxamic acid was reported to show a rapid degradation rate in the presence of certain types of ruminal microbiota, and in some cases causing 100% AHA degradation within 24 h of fermentation [8,9]. Therefore, there is a need to discover new effective urease inhibitors which are safe and stable in the presence of microbiota. In the past decades, a variety of compound libraries have been screened by molecular docking to find lead molecules for urease inhibitors [10]. Few of them were targeting ruminal microbiota. Therefore, it is important to find lead molecules specifically targeting ruminal microbiota.

Molecular docking involves computationally screening large databases for compounds that could serve as a potential inhibitor of the target protein structure [11]. This approach dramatically reduces the number of candidate compounds to be tested biologically. Molecular docking relies on the structure information of the target urease structure to virtually screen the specific compound database. The previous molecular docking studies were targeted at pathogens such as *Proteus mirabilis*, *Ureaplasma urealyticum*, *Helicobacter pylori,* and *Klebsiella aerogenes*, which were thought to be causative factors of many clinical conditions [12]. These docking studies have helped find a range of compounds including many quinolones, heterocyclic compounds, organophosphorus compounds, flavones, Schiff bases, and different coordination complexes, which enriched the diversity pool of urease inhibition scaffolds [13]. However, none of them were targeting ruminal urease and to date the experimentally derived protein structure for urease of rumen microbial origin remains unknown, which hinders the design and construction of novel urease inhibitors specifically targeting ruminal microbiota. Homology modeling could construct an atomic-resolution model of an unknown protein structure to facilitate molecular docking when the three-dimensional structure of the target is not available.

In general, the urease active site of bacterial ureases is located at the UreC coded protein area, most of which contains two nickel ions bridged by a carbamylated lysine and coordinated with some other amino acid residues [14]. Apart from the urease active site, the flap region covering the active area is recognized as an important allosteric site, which is highly influential for the position of the flap and the urease activity. The cysteine residue located on the flap region can regulate the position and flexibility of the flap, so it is likely that cysteine could be used as a targeting point to inhibit the urease activity [15].

In a previous study, our research group found a ruminal metagenomic urease gene cluster from the ruminal microbial metagenome [16]. In continuation of our efforts to study the urease of rumen origin as a potential target for better regulation of rumen microbiota related urease activity, an integrated chemical database screening strategy was employed involving ruminal metagenomic urease gene cluster based homology modeling and molecular docking, with the aim of finding urease inhibitors specifically targeting urease producing bacteria in ruminal microbiota.

## 2. Results

### 2.1. Ruminal Metagenomic Urease Homology Modeling and Validation

The UreC (567 aa) of ruminal microbial metagenomic urease (GeneBank_ID of MN660246-MN660252) was highly homologous with the *Klebsiella aerogenes* urease (PDB_ID: 4EP8) with a 69.59% sequence identity. The GMQE and QMEAN values of the homology model were 0.85 and −0.37, respectively. The GMQE combines properties from both the target-template alignment and the template search method to evaluate the modeling result. Its number ranged between 0 and 1, reflecting the expected accuracy of a homology model and higher number indicating higher reliability. The QMEAN around zero indicated a good agreement between the model structure and experimental structures of similar size, whereas scores of −4.0 or below were indications of models with low quality. Both parameters measured here indicated a good modeling quality of the UreC region of ruminal microbial metagenomic urease.

**Figure 1:**
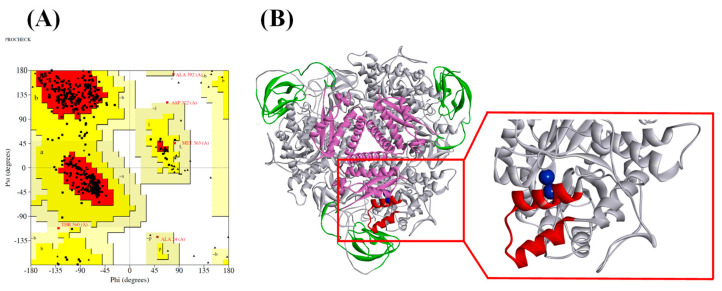
Ramachandran plots (**A**) and final three-dimensional (3D) structure of the ruminal metagenomic urease (UreC) homology model (**B**). Subunits of the ruminal metagenomic urease homology model are indicated by a different color; the trimer of alpha subunits (UreC) is depicted as grey, the beta subunits (UreB) as green, and the gamma subunits (UreA) as pink. The UreC structure of ruminal metagenomic urease is magnified in the rectangular window in which Ni pairs are shown as blue spheres and the flexible loop is depicted in red.

The quality of the three-dimensional (3D) model was further evaluated via a Ramachandran plot using the PROCHECK software (Figure 1A). It revealed that 424 amino residues (89.6%) were in the most favorable region, 44 amino acids (9.3%) were in the allowed region, and only one amino acid (0.2%) was in the disallowed region. This indicated that the constructed model was of good quality, with an ERRAT value of 96.9371. For the Verify 3D value, the server predicted that 89.38% of the residues in the model had an average 3D-1D score > 0.2, indicating a good quality of the constructed model.

The spatial model of the ruminal metagenomic urease consists of alpha subunits (UreC), beta subunits (UreB), and gamma subunits (UreA), which form (αβγ)_3_ trimers (Figure 1B). The active site of the homology model was revealed by sequence alignment between *Klebsiella aerogenes* urease and ruminal metagenomic urease, which consists of Lys 216, His 218, His 245, His 271, Gly276, His 133, His 135, and Asp 359 (Figure S1). All these residues were in the most favorable region of the Ramachandran plot. The Lys 216 in the model may serve as a bridge to connect two nickels in the active site. In addition to the amino acid residues directly involved in the construction of the active site, the residues comprising the mobile flap located outside of the active site were also playing an important role in the urease catalysis function, by stabilizing the catalytic transition state and accelerating the reaction, the flap thought to be acting as a gate for the substrate. Reports of virtual screening targeting this flap area resulted in some good candidates for urease inhibitors [15]. The flap area of ruminal urease ranged from lLe 308 to lLe 336 (Figure 1B). This fragment was considered for virtual screening.

### 2.2. Virtual Screening for Candidate Compounds

After the ChemDiv database preparation, a total of 0.87 million compounds were filtered out according to the modified Lipinski’s rule of five. The first round of virtual screening produced 8753 hits. After the second round of screening, 20 compounds were selected as candidate compounds for the urease activity experiment against rumen microbial urease, with a top ten docking score shown in Table 1. The docking scores for top 11–20 compounds are provided in Supplementary Table S1.

### 2.3. Urease Enzyme Inhibition Rate, IC_50_ Value, and Inhibition Mode of Compounds

**Figure 2:**
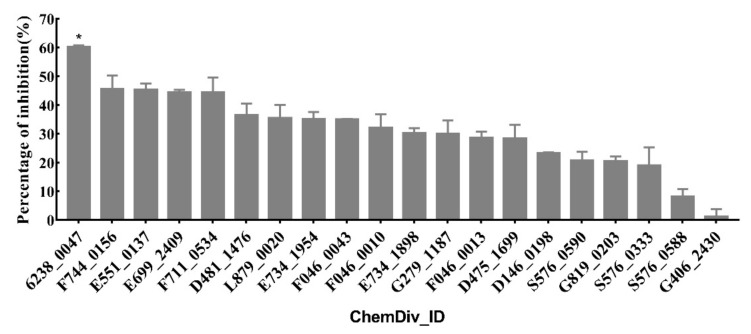
Percentage of inhibition of candidate compounds (0.5 mM) against ruminal microbial urease. Asterisks (*) indicate that the difference between compound 6238-0047 and other candidate compounds were significant (*p* < 0.05).

The percentage of inhibition for each compound is shown in Figure 2. Compound 6238-0047 showed a superior ruminal microbial urease inhibition capacity.

**Figure 3:**
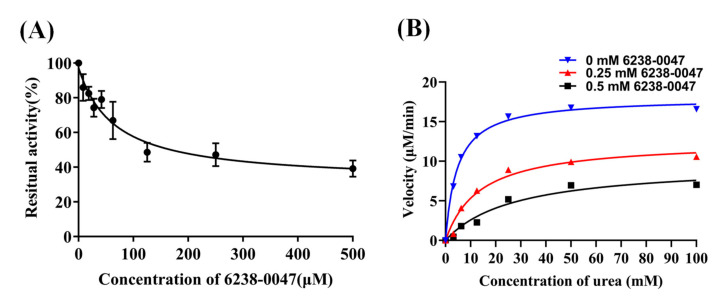
IC_50_ identification (**A**) and kinetic characterization (**B**) of compound 6238-0047 against ruminal microbial urease.

The IC_50_ value of compound 6238-0047 was determined to be 65.86 μM, which was superior to the reference inhibitor acetohydroxamic acid with an IC_50_ value of 158 μM measured at the same condition (*p* < 0.01). The R-square of the nonlinear regression curves for compound 6238-0047 and acetohydroxamic acid were 0.9622 and 0.9826, respectively. Kinetic studies showed that the Michaelis-Menten kinetic parameters Km and Vmax changed with the addition of different concentrations of compound 6238-0047 (Figure 3B). Specifically, the addition of only the solvent of compound 6238-0047 had an enzyme kinetic curve with Km value of 4.630 mM, Vmax value of 18.00 μM/min, and Vmax/Km value of 3.8877. The 0.25 mM of compound 6238-0047 had an enzyme kinetic curve with Km value of 13.61 mM, Vmax value of 12.58 μM/min, and Vmax/Km value of 0.9243. The 0.5 mM of compound 6238-0047 had an enzyme kinetic curve with Km value of 27.39 mΜ, Vmax value of 9.67 μM/min, and Vmax/Km value of 0.3529.

The Km and Vmax values of the rumen microbial urease kinetic curve have changed simultaneously with the treatment of different concentrations of compound 6238-0047 and the parameter Vmax/Km also changed, indicating that the small molecule compound 6238-0047 was a mixed-type ruminal microbial urease inhibitor.

### 2.4. Inhibition Capacity of the Compound on Ruminal Microbial Community

**Figure 4:**
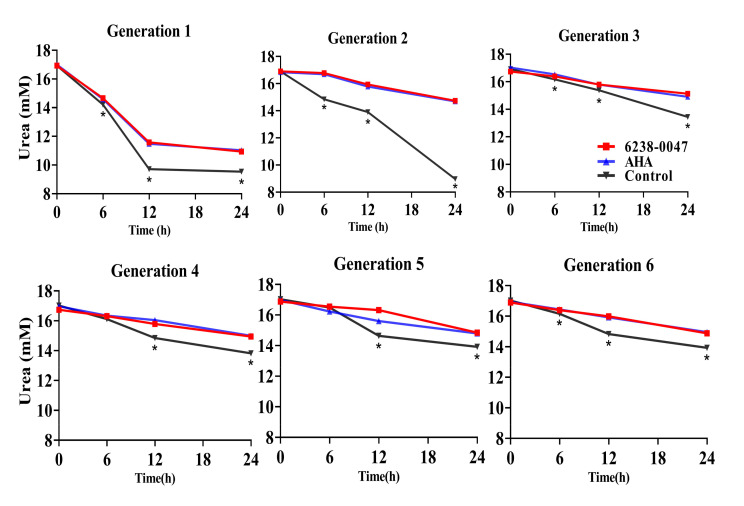
Influence of compound 6238-0047 on the urea in rumen fermentation in vitro. Each point represents the mean of three observations. Acetohydroxamic acid (AHA) acted as a positive control, and the control group only added the methanol solution. Asterisks (*) indicate that the difference between the control group and compound treated group (AHA and 6238-0047) was significant (*p* < 0.01).

The changes in urea concentration over the course of 24 h of incubation for six generations of ruminal microbial fermentation are illustrated in Figure 4. For the first two generations, the urea decreased more rapidly than the following generation. For each generation, the addition of AHA and compound 6238-0047 significantly lowered the amount of urea degradation compared with the control group for all time points in every generation (*p* < 0.01) except at the six hour in the fourth and fifth generation.

**Figure 5:**
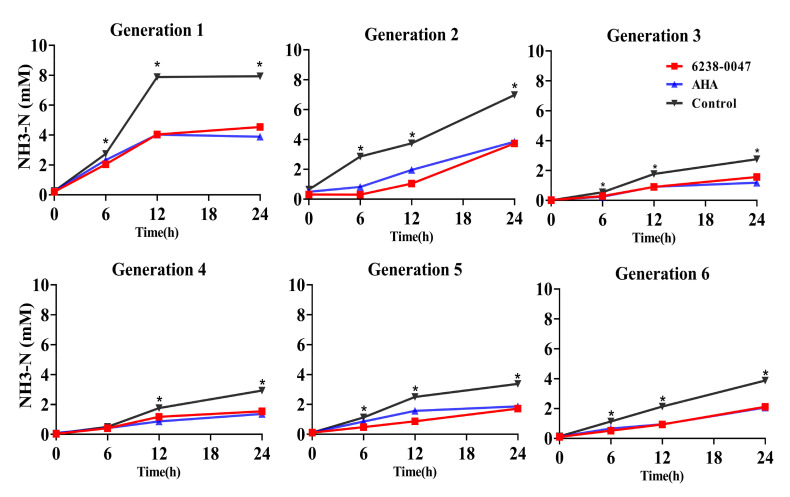
Influence of compound 6238-0047 and AHA on the amount of NH3-N production in rumen fermentation in vitro. Each point represents the mean of three observations. Acetohydroxamic acid (AHA) acted as a positive control, and the control group only added the solvent. Asterisks (*) indicate that the difference between the control group and compound treated group (AHA and 6238-0047) was significant (*p* < 0.01).

The decrease in urea degradation simultaneously reduced the amount of NH3-N production in the system (Figure 5), in which the concentrations of NH3-N in the AHA and compound 6238-0047 group were significantly lower than the control group for all time points in every generation (*p* < 0.01) except at the six hour in the fourth generation.

**Figure 6:**
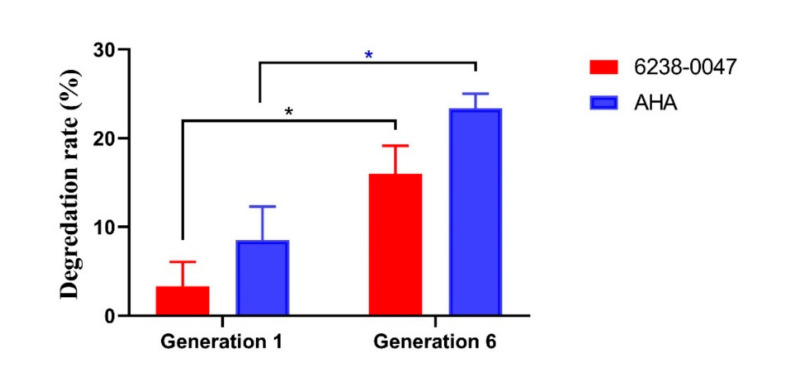
Chemical degradation rates of AHA and 6238-0047. The results were presented as the mean ± SEM. Asterisk (*) indicates the significant difference.

The presence of AHA and compound 6238-0047 was revealed by the chemical degradation rates of AHA and compound 6238-0047 (Figure 6). In the first-generation ruminal microbial culture, the 24 h degradation rate of AHA and compound 6238-0047 were 8.54% and 3.32%, respectively, compared with the sixth generation ruminal microbial culture of 23.39% and 16.00%, respectively.

### 2.5. Toxicity Valuation of Screened Compound

**Figure 7:**
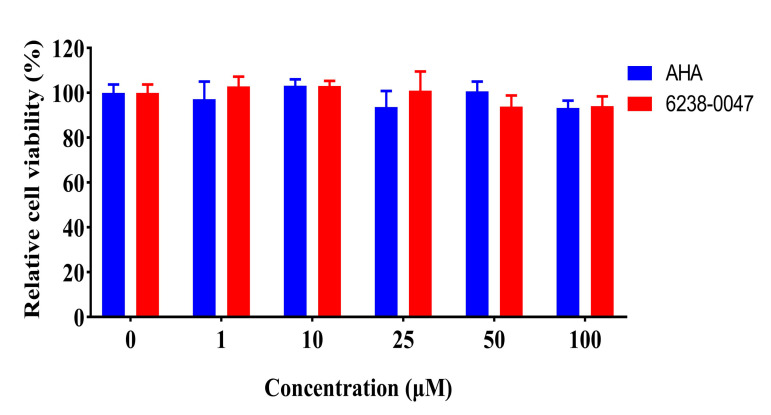
Influence of AHA and compound 6238-0047 on cell viability of Caco-2 cell culture. The results were presented as the mean ± SEM.

The intestinal tract is the place where the nutrients such as amino acids and glucose are absorbed and they are more likely to be compromised by the toxicity effect of small molecular compounds. Therefore, the toxicity effect of compound 6238-0047 against intestinal cells needed to be evaluated. Caco-2 cells can differentiate into enterocytes with features of the small intestine and are currently one of the most used cell lines for the evaluation of the toxicological effect of compounds, so it was used in this study to evaluate the toxicity of compound 6238-0047. The results indicated that compound 6238-0047 and AHA had no significant influence (*p* > 0.05) on the relative cell viability of Caco-2 cell culture with their concentration below 100 μM (Figure 7), indicating the noncytotoxic property of compound 6238-0047.

### 2.6. Chemical Information and Binding Mode

**Figure 8:**
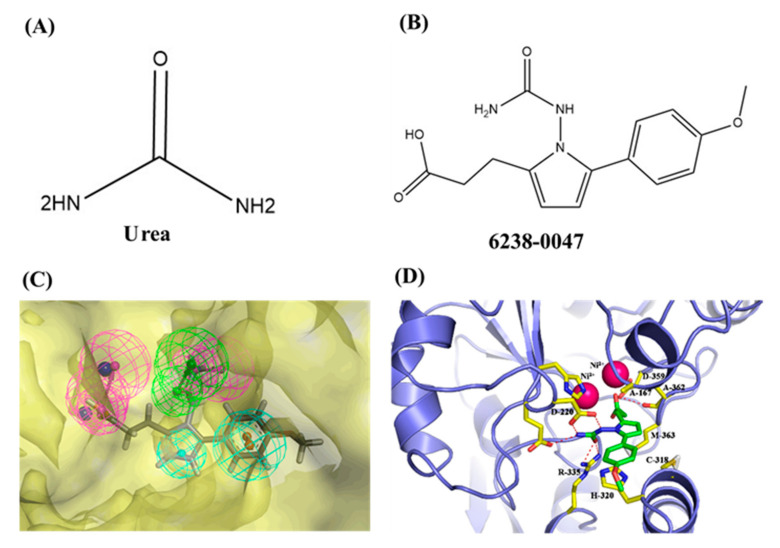
Chemical information and binding mode of compound 6238-0047. The structural formula and the chemical name of urea (**A**)and compound 6238-0047 (**B**). The structure properties of compound 6238-0047 were verified by the receptor-ligand pharmacophore between compound 6238-0047 and the ruminal metagenomic urease active site (**C**). Binding mode of compound 6238-0047 with the active site of the ruminal metagenomic urease homology model was revealed by molecular docking (**D**). The surface of the active site pocket and the nickel ions are shown in transparent yellow color and blue color, respectively. The web spheres in green, light blue, and rose red indicate the hydrogen bond acceptor, hydrophobic fragments, and hydrogen bond donor, respectively. The chemical structure of compound 6238-0047 was superimposed to its pharmacophore in grey color. The active site is colored in light purple color while its critical amino acid residues are represented by a yellow stick. Compound 6238-0047 is depicted in green color, while their hydrogen-bond-related functional groups are painted in red and blue, respectively. The red dotted lines indicate where the hydrogen bonds formed. Two nickel atoms are demonstrated in rose color.

The name of compound 6238-0047, a novel urease inhibitor found in this study, is 3-[1-[(aminocarbonyl)amino]-5-(4-methoxyphenyl)-1H-pyrrol-2-yl] propanoic acid, the structure of which is shown in Figure 8B. The propionic acid group in the chemical structure of compound 6238-0047 was oriented at the active site, hence being defined as the front part of the chemical structure. Compound 6238-0047 also contains a urea-like group in the upper-middle part, and an anisole group in the tail part of the chemical skeleton. These three distinctive chemical groups were bridged by a pyrrole ring in the middle. Urea is a natural substrate of urease (Figure 8A). Compounds containing a fragment of urea are favored for the construction of inhibitors of urease and many compounds have been screened bearing the urea structure [17,18], so the binding ability of this urea analogue fragment to the active site could be explained.

The binding mode of compound 6238-0047 was revealed by molecular docking (Figure 8D). This figure is also provided in Figure S2. Three hydrogen bonds were formed between the urea-like group of compound 6238-0047 and the active site residues of Arg335 (one hydrogen bond, distance: 2.28 Å) and Asp220 (two hydrogen bonds, distance: 1.96 and 2.07 Å). Two hydrogen bonds were also formed between the carboxyl group of compound 6238-0047 and the important active site residues of Ala362 (one hydrogen bond, distance: 2.73 Å) and Asp359 (one hydrogen bond, distance: 1.93 Å). The Pi-Alkyl interaction was formed between Cys318 and the middle pyrrole part of compound 6238-0047. A relatively strong hydrophobic interaction was formed between compound 6238-0047 and His320, Met363, and Ala166.

The property and the fitting position were also visualized by the receptor-ligand pharmacophore (Figure 8C). The lateral view of the surface of the active site pocket and compound 6238-0047 suggested that the compound fitted well into the urease active site pocket. The results demonstrated that the pharmacophore possessed six chemical characteristics including two hydrogen bond acceptors (HBA), one hydrophobic group (HY), two hydrogen bond donors (HBD), and one aromatic ring (RA).

## 3. Discussion

Hydroxamic acids have been the best recognized urease inhibitors, and they have been widely used as a reference urease inhibitor for the development of novel urease inhibitors [19]. It is the only commercially available medical urease inhibitor with an IC_50_ value of 18.2 μM against *H. pylori* urease [20]. However, the IC_50_ value of AHA changes when it comes against different sources of urease. For the jack bean urease, the IC_50_ value of AHA could be changed to 60 μM [21]. This is also evident for other urease inhibitors, which hold different IC_50_ values and inhibition types against different sources of urease [22], which makes it necessary to find a urease inhibitor specifically targeting the ruminal microbial urease. In this study, compound 6238-0047 was virtual screened specifically to target the ruminal metagenomic urease detected in the samples of rumen origin and it showed a superior IC_50_ value than the reference urease inhibitor AHA against rumen microbial urease. The superior IC_50_ value can be explained by the fact that it can fit right into the active site pocket of ruminal microbial urease and was interacting with its flap loop outside the active site to inhibit its activity. This may put compound 6238-0047 at an advantage against rumen microbial urease compared to AHA.

Studies of enzymes were often conducted against pure enzymes, but the urease from the rumen was of multiple origins, which makes it of no practical value to study the effect of screened compounds on urease such as Jack bean urease. Therefore, this study uses rumen microbial urease, as a more pragmatic and realistic situation.

The Lineweaver-Burk analysis is a common method of linearizing substrate-velocity data to determine the kinetic constants Km and Vmax, but it transformed the original data in a double reciprocal manner and this caused the amplification of the error. Some experimental points with higher substrate concentrations may cluster together near the intersection of axes after the transformation and was not suitable for the subsequent linear curve fitting [23,24]. Nonlinear curve fitting can keep the original data by plotting the substrate concentration directly against velocity with no secondary transformation of the data and this could be achieved by using the GraphPad Prism software, and the relevant parameter such as R-square value, Km, and Vm can be calculated automatically after the nonlinear curve fitting, so this calculation method was employed in this study.

Ludden et al. [25] had used different concentrations of N-(n-butyl) thiophosphoric triamide (NBPT) as treatments to study the effect of this compound on rumen ammonia nitrogen production. It was found that on the second day of the treatments to cows with a permanent rumen fistula. The amount of ruminal ammonia nitrogen production under each compound concentration was lower than that of the control group. However, on the fifteenth day of the treatments, the amount of ruminal ammonia nitrogen production under each compound concentration tended to approach the control group with ruminal ammonia nitrogen production in some groups of lower compound concentration at an even level with the control group, indicating that with the increase of the treatment days of the inhibitor, rumen microbiota had become adaptable to NBPT. The adaptability of rumen microbiota to the urease inhibitors is one of main concerns for developing a novel urease inhibitor. For compound 6238-0047, there was little difference between inhibitors, treatments, and controls in the amount of urea degradation through the first six hours of incubation. This initial small decrease in urea and NH3-N concentrations for all treatments may indicate a lag time before the maximal effect of AHA and 6238-0047. Such phenomenon was also observed in other traditional urease inhibitor reports. The curve trend of NH3-N production for compound 6238-0047 was consistently lower than the control group, indicating no sign of microbial adaptability for the compound.

The degradation of AHA inside rumen fluid has been widely reported and the rate of degradation varied among different strains of bacteria derived from rumen fluid [9]. For some strains and rumen fluids, AHA may completely disappear in the in vitro fermentation system within 24 h [8,9], revealing that the ruminal microbiota has a compound degradative capacity. The degradation rate of AHA increased in the sixth generation, which indicated that the compound degradative capacity of ruminal microbiota increased and may suggest a shift in the balance of rumen microbiome. A comparison between compound 6238-0047 and AHA showed that the former was relatively more stable than the latter in the same fermentation system.

The crystal structure of *Klebsiella aerogenes* urease revealed that the nickel containing active site of urease was located in domain 1 (residues α130 to α414) of the α-subunit (ureC coding), and it contained a binuclear nickel (Ni) center, bridged by a carbamylated lysine through its O-atoms, with the first Ni site further coordinated by two histidines through their N-atoms, and the second Ni site by two histidines through N-atoms, but also by aspartic acid through its O-atom [26]. The site mutants study also confirmed that carbamylated Lys 217 and His219 were crucial for an active site function [27]. The active site of the template was extracted from the PDB file of the *Klebsiella aerogenes* urease (PDB_ID: 4EP8). It consisted of Lys 217, His 219, His 246, His 272, Gly277, His 134, His 136, and Asp 360. The active site of ruminal metagenomic urease was revealed by a sequence alignment with the *Klebsiella aerogenes* urease. It consisted of two nickel ions, bridged by Lys216, with one nickel bound to His 133 and another nickel bound to His 245. His 271 and Asp 359 also coordinate to one of the nickel ions (Figure S1). Compound 6238-0047 could form a direct hydrogen bond with Asp359 and interact with some of the other residues near nickel ions, indicating that it could insert itself into the active site. Previous studies on the mechanism of urease inhibition suggested a mobile protein flap region near the active site as an important allosteric site for the inhibition of urease activity [22]. Such a flap region for the active site of the ruminal metagenomic urease ranged from ILe 308 to ILe 336 and the Cys318 site in this region acted as a ligand stabilizing residue [15]. Compound 6238-0047 could interact with Cys318 to interfere with the normal function of the active site flap. All these binding modes showed that compound 6238-0047 could not only bind and interact with residues in the active site pocket but also with the flap outside the active side and these binding behaviors often result in a mixed type inhibitor in the previous report [28,29,30] and the kinetic study of compound 6238-0047 confirmed that it was a mixed type inhibitor.

The structure of this newly found urease inhibitor is relatively large compared with the reference inhibitor AHA. The branched structure of urea motif and (CH2CH2COO-) fragment containing carboxyl group, and the heterocyclic group and the phenol fragment may be considered unfavorable for a chemical to pass through the flap area and fit into the active site pocket of urease. However, recent studies have shown that urease has a large active site pocket and is flexible enough to bind to relatively larger chemical structures [31]. The flap is located outside of the urease active site and it functions as a gate for substrate ingress and product egress of urease, so the urease activity is tightly controlled by an active site flap. The open states and closed states of urease flap have been well characterized and a previously unobserved wide-open flap state has been reported [32]. A previous report also pointed out that the closed flap region of the binding pocket is solvent exposed, which acts as a substrate/product reservoir. This study showed that the relatively large small-molecule-compound had the chance to enter the urease active site pocket and inhibit the urease activity. For compound 6238-0047, a relatively larger scaffold than the reference inhibitor AHA may not hinder its way into the active site pocket and more variety of the fragment inside the chemical structure adds additional binding sites for compound 6238-0047, hence boosting the inhibitory ability of this compound against rumen microbial urease.

## 4. Materials and Methods

### 4.1. Homology Modeling of Rumen Metagenomic Urease

The urease amino acids from ruminal microbial metagenome (GeneBank_ID of MN660246-MN660252) were submitted to the NCBI Blastp server (https://blast.ncbi.nlm.nih.gov/Blast.cgi) to find the template protein with a relatively intact crystal structure and high similarity in the RCSB Protein Databank. The three-dimensional structure of the UreC of ruminal metagenomic urease was modeled using the SWISS-MODEL server with default parameters [33]. The resulting model quality was preliminarily estimated by two parameters provided by SWISS-MODEL: Global Model Quality Estimate (GMQE) and the QMEAN scoring. The quality of the model was verified using the Structural Analysis and Verification Server (SAVES 5.0) (http://servicesn.mbi.ucla.edu/SAVES/), which contains a wide range of software and algorithms such as PROCHECK for checking the stereo chemical quality of the protein structure, ERRAT for checking noncovalently bonded amino acid interactions, and VERIFY 3D for using amino acid sequence to assess the compatibility of the protein structure. The active site of the homology model was evaluated manually by superimposing it with the active site of the template protein. The tertiary protein structures of the UreABC of ruminal metagenomic urease were also reconstructed for visualization by using the multiple template homology modeling of the SWISS-MODEL [33].

### 4.2. Molecular Docking-Based Virtual Screening for Urease Inhibitors

The homology model, which contains the α308 to α336 flexible flap and the active site, was selected as a target protein structure, forming an active site pocket composed by these amino acid residues with two nickel ions. All the water molecules and co-crystallized ligands were removed. Protein hydrogenation was also conducted. The ILe 308–ILe 336 region was selected to generate the active site pocket for the molecular docking by the protomol module of Surflex-dock, which based on “Residues” mode, the threshold parameter was set to 0.5 and bloat was set to the default value. The coordinates for grid center of the active site pocket was revealed by the Discovery studio visualizer (Accelrys Software Inc., San Diego, CA, USA) as 56.527 Å X −18.581 Å X 28.727 Å.

The Chemdiv Compound Libraries (ChemDiv, San Diego, CA) provide a shelf-available set of over 1.5 million compounds. The “compound filtering” module of the Sybyl-X2.1 software was used to retrieve compounds from the ChemDiv Compound Libraries according to the slightly modified Lipinski’s rule of five to find compounds that have drug-like properties, namely molecular weight <700, partition coefficient logP within the range of −4 and 6, number of H-bond donors < 6, number of H bond acceptors < 15, and number of rotatable bonds <10 [34].

Two rounds of virtual screening were implemented using the Surflex module of the Sybyl-X2.1 software. In the first round, the default settings were altered by changing the selection of the default “per-dock minimization” and “post-dock minimization” to canceled, “max conformations per fragment” was changed from 20 to 10, “max number of rotatable bonds per molecule” was changed from 100 to 50, and “maximum number of poses per ligand” was changed from 10 to 3 to limit the number of the conformation of the compounds to expedite the virtual screening. In the second round of virtual screening, all default parameters were restored to original settings to further screen compounds produced in the first round. Detailed procedures for virtual screening are also provided in Supplementary Materials.

### 4.3. Ruminal Microbial Urease Preparation

Rumen fluid samples were collected from five Chinese Holstein dairy cows with permanent rumen fistula 3 h after feeding a total mixed ration in the morning. All the operations were conducted in accordance with the protocol approved by the Animal Care and Use Committee for Livestock of the Institute of Animal Sciences, Chinese Academy of Agricultural Sciences (Permit Number: IAS2019-14, Permit Date: 22-03-2019). All rumen fluid samples were filtered by a six layer sterilized medical gauze and mixed in a sterilized beaker before being evenly distributed into different 50 mL tubes and stored in liquid nitrogen. The deforested samples were centrifuged at 12,000× *g* for 10 min at 4 °C, then the supernatant was removed from the tube. The remaining sediments were resuspended in a HEPES buffer solution (pH 7.5, 50 mM) and then centrifuged at 12,000× *g* for 10 min at 4 °C. The supernatant was discarded and the remaining rumen bacteria were collected and stored at −80 °C for later urease extraction. All the sample handing processes were conducted on an ice bath. Before each urease inhibition assay, 2 g of rumen bacteria were taken from the fridge and resuspended in a HEPES buffer followed by lysis through a high-pressure cell press JG-1A (Ningbo Scientz Biotechnology Co., Ltd., Ningbo, China). The hollow cylindrical sample loading element was placed in −80 °C for 10 min before the high-pressure lysis at 22,000 psi. The subsequent fluid was centrifuged at 12,000× *g* for 10 min at 4 °C to obtain the supernatant as ruminal microbial urease.

### 4.4. Urease Activity Inhibition and Kinetic Characterization

In vitro urease inhibition screening assays were conducted to further narrow the virtual screened compounds for further IC_50_ determination and kinetic characterization. The urease inhibition assay was performed with slight modifications of the Berthelot alkaline phenol-hypochlorite method [35]. Candidate compounds filtered by virtual screening were diluted in a methanol solution. The assay mixture containing 15 μL of ruminal microbial urease and 15 μL of the candidate compounds with 70 μL of urea solution (50 mM, 0.15 g urea diluted in a HEPES buffer (pH 7.5, 50 mM)) was incubated in a water bath at 37 °C for 30 min. The final concentration for the candidate compounds in the assay mixture was 0.5 mM. The ammonia that was produced was measured using 50 μL of a phenol reagent containing 10.0 g/L phenol and 50 mg/L of sodium nitroprusside, and 50 μL of alkali reagent containing 5 g/L sodium hydroxide and 8.4 mL/L of sodium hypochlorite solution, at 37 °C for 30 min. The absorbance of each well was measured at 625 nm by a micro plate spectrophotometer (Thermo Scientific Varioskan Flash G-282, Thermo Scientific, Waltham, MA, USA). The endogenous nitrogen (blank) was measured by adding a 70 μL HEPES buffer (pH 7.5, 50 mM) containing no urea and 20 μL methanol solution in the first 30 min of the reaction mixture. The 100% initial activity was determined by adding a 15 μL methanol solution in the first 30 min of the reaction mixture. The urease inhibition (%) was calculated by the following formula:

$$\mathrm{Urease}\;\mathrm{inhibition}\left(\%\right)=\left(1-\frac{a-c}{b-c}\;\right)\times 100$$

where a is the absorbance of inhibitory well, b is the absorbance of 100% initial activity without an inhibitor, and c is the absorbance of the endogenous nitrogen (blank). All the assays were performed in triplicate.

One compound was selected for further determination of the IC_50_ value and inhibition mode based on the most potent inhibition potency at the concentration of 0.5 mM. Different concentrations of the compound were added to the assay mixture at 500, 250, 125, 62.5, 41.7, 27.8, 18.5, 8.2, and 0 µM final concentrations for determination of the IC_50_ value. The IC_50_ value of acetohydroxamic acid was also tested at the same series of concentrations as a reference. The residual activity of the addition of each concentration of the compounds was calculated by the following formula:

Residual activity (%) = 100 − Urease inhibition (%)

The IC_50_ value was calculated by plotting each concentration of urease inhibitor against the corresponding residual activity of the enzyme using the nonlinear regression curve fitting function of GraphPad Prism, version 8.0.1 (GraphPad Software, San Diego, CA, USA).

The kinetic of the compound was determined by varying the concentration of urea in the presence of different compound 6238-0047 concentrations of 0.25 and 0.5 mM in the assay mixture. The control group only added the solvent of compound 6238-0047. The concentrations for the substrate urea varied between 100, 50, 25, 12.5, 6.25, and 3.125 mM in the assay mixture, and the procedure for measuring urease activity was the same as the urease inhibition assay. Maximal initial velocities were determined from the initial linear portion of the absorbance up to 10 min after the addition of the enzyme.

Kinetic parameters Km and Vmax were calculated by the nonlinear fitting of different substrate concentrations against the velocities of the reaction at each different concentrations of compound 6238-0047 using the GraphPad Prism software as mentioned before.

### 4.5. Evaluation of Screened Compound by Ruminal Microbial Fermentation

Seven solutions were prepared for the anaerobic media as solution A (3 g/L K_2_HPO_4_), solution B (0.6 g/L CaCl_2_, 3 g/L K_2_HPO_4_, 6 g/L NaCl, 0.6 g/L MgSO_4_·7H_2_O), solution C (6 g/L K_2_HPO_4)_, solution D (1.6 g/L CaCl_2_·2H_2_O, 6 g/L K_2_HPO_4_, 12 g/L NaCl, 2.5 g/L MgSO_4_·7H_2_O), trace element solution (300 mg/L H_3_BO_3_, 100 mg/L ZnSO_4_·7H_2_O, 30 mg/L MnCl_2_·4H_2_O, 20 mg/L CoCl_2_·6H_2_O, 30 mg/L Na_2_MoO_4_·2H_2_O, 10 mg/L Na_2_SeO_3_, 20 mg/L NiCl_2_, 10 mg/L CuCl_2_·2H_2_O, 150 mg/L FeCl_2_·4H_2_O), volatile fatty acid (VFA) mixed solution (17 mL acetic acid, 6 mL propionic acid, 4 mL butyric acid, 1 mL n-valeric acid, 1 mL isovaleric acid, and 1 mL isobutyric acid), and hemin solution, prepared by diluting 50 mg of Haemon in NaOH (1 N solution) and made up to 100 mL with distilled water.

Each liter of anaerobic media contained 30 mL of autoclaved rumen fluid supernatant, 0.5 g of glucose, 0.5 g of soluble starch, 0.5 g of cellobiose, 0.5 g of maltose, 0.5 g of amicase, 0.5 g of tripticase, 0.5 g of yeast extract, 8 g of NaHCO_3_, 0.1 mL of trace elements solution, 15 mL solution A, 15 mL solution B, 0.5 g of cysteine hydrochloride, 10 mL of hemin solution, 3.1 mL of VFA mixed solution, and 1 mg resazurin.

The anaerobic urea solution was prepared using an anaerobic glove box, by dissolving 2.5 g of urea in a 50 mL anaerobic diluent. This diluent consisted of 3.8 mL of salt solution C, 3.8 mL of salt solution D, 5 mL of 8% sodium Na_2_CO_3_, 1 mL of 0.1% resazurin, 0.5 g of cysteine hydrochloride dissolved in distilled water and made up to 1000 mL and distributed into 50 mL anaerobic bottles. All the anaerobic bottles were sterilized by autoclave. Prior to the inoculation, fresh frozen rumen fluid samples, pretreated as described previously, were removed from the freezer, thawed, homogenized, and used immediately.

The generation fermentation experiments were performed in anaerobic tubes containing 5 mL of an anaerobic medium, and in total, six generations of fermentation were performed. Altogether 54 tubes were prepared with nine tubes for each generation of fermentation. For each generation, nine tubes were separated into three groups as the AHA treated group, compound 6238-0047 treated group, and control group with each group containing three tubes. For the AHA treated group, the AHA working solution was prepared by diluting 3.76 mg AHA with 10 mL of methanol (5000 μM AHA). For the compound 6238-0047 treated group, a compound 6238-0047 working solution was prepared by diluting 15.166 mg of compound 6238-0047 with 10 mL of methanol (5000 μM of compound 6238-0047). For the control group, the working solution contained only the solvent (methanol). All the working solutions were filtered by 0.22 μm of a membrane filter (Millipore Express PES Membrane, MILLEX GP). For each group, 100 µL of the working solution was added to each tube to mix with the anaerobic medium. The final concentration for AHA and compound 6238-0047 in the anaerobic medium of a tube was 100 μM.

Before the inoculation of each tube in each generation with fresh rumen fluid, 100 µL of the anaerobic urea solution was extracted from the anaerobic bottle using a syringe and filtered through 0.22 μm membrane filters, and then injected to the anaerobic tube to make the urea concentration at 16.65 mM in the tube. Then, 100 μL of AHA, compound 6238-0047, and methanol were filtered through 0.22 μm membrane filters and injected into each tube respectively, with the concentration of compound 6238-0047 and AHA at 100 μM. In the first generation, 100 µL of fresh rumen fluid was inoculated using a syringe, the generations thereafter were inoculated with the inoculum from the previous generation. All tubes were kept in the dark at 39 °C for 24 h.

A 200 μL sample of culture fluid were taken from each tube at 0, 6, 12, and 24 h of every generation. After centrifugation at 12,000× *g* for 5 min, 180 μL of supernatant was taken to mix with 1.8 μL of 8 mol/L HCL and then stored at −25 °C for the determination of NH4-N and urea-N. At 0 h of each generation, a 200 μL sample was taken and centrifuged at 12,000× *g* for 5 min to collect the supernatant for compound concentration determination. At 24 h for each generation, a 1 mL sample was taken from each tube and centrifuged at 12,000× *g* for 5 min. A sample of 500 μL of supernatant was used for the determination of the concentration of the compound. The NH4-N and Urea-N concentration was measured by the Berthelot alkaline phenol-hypochlorite method [35] and the diacetyl monoxime method kit (Nanjing Jiancheng Co., Nanjing, China) following the manufacturer’s protocol. The content of compound 6238-0047 and AHA were measured by the ultra-performance LC system (Agilent 1290 Infinity II, Agilent Technologies, Waldbronn, Germany) and high resolution mass spectrometry (5600 Triple TOF Plus, AB Sciex, Singapore) equipped with an ESI source. Chromatographic separation was performed on a reversed-phase ACQUITY UPLC HSS T3 1.8 μm, 3.0 × 100 mm columns (Waters, Dublin, Ireland). Chromatographic gradients were referenced to the paper [36]. Mass spectrometry was performed using a high resolution mass spectrometer (5600 Triple TOF Plus, AB Sciex, Singapore) equipped with an ESI source. Quantification for compound 6238-0047 and AHA in different samples was done using standard calibration curves and the degradation rate of the compound was then calculated.

### 4.6. Toxicity Evaluation of Screened Compound Using a Caco-2 Cell Culture

A standard CCK8 colorimetric assay was employed for the evaluation of cytotoxic activity of compound 6238-0047 using 96-well flat bottomed microplates. Dulbecco’s Modified Eagle Medium (GIBCO) with 10% FBS (fetal bovine serum, FBS, Gibco) and 1% NEAA was used for the culturing of Caco-2 cells (ATCC, Manassas, VA, USA), and the medium was supplemented with 100 μg/mL of streptomycin and 100 IU/mL of penicillin. The medium was kept in 5% CO_2_ and incubated at 37 °C. The exponentially grown cells were harvested, counted using a hemocytometer and diluted with the medium. The cell culture was diluted to a density of 1 × 105 cells/mL and transferred into a 96-well plate with 135 μL or 5000 cells per well. The medium was incubated for 24 h for cell attachment. Following this, 15 μL of compounds with different final concentrations of 1, 10, 25, 50, and 100 μM in the experimental group and fresh medium as a control group, were added to a plate with five replicates for each. After 24 h of incubation at 37 °C and kept in 5% CO_2_, 15 μL of CCK8 was added to each well and after 2 h the absorbance was measured at 450 nm using a microplate reader. The formula for calculating the relative cell viability was as follows:

Relative cell viability (%) = absorbance of experimental group/absorbance of control group × 100%

### 4.7. Visulization of the Docking Position of the Screened Compound against Urease

The virtual screening by the Sybyl-X2.1 software produced a molecular docking result for compound 6238-0047 with the homology model. This complex was inputted into the PyMOL software for visualization of the binding mode. Compound 6238-0047 was further characterized by a receptor-ligand pharmacophore.

### 4.8. Statistical Analysis

Nonlinear curve fitting of the activity of rumen urease under different concentrations of compounds was conducted by GraphPad prism, version 8.0.1 (GraphPad Software, San Diego, CA, USA) to obtain the IC_50_ value of compounds and the kinetic curve of urease, and the data points were presented as the mean ± SEM. The differences between compound treatment groups and the control on urea concentration and ammonia concentration in each generation or time were analyzed using the ANOVA procedure of SAS (SAS Institute Inc., Cary, NC, USA) and *p* < 0.05 indicated a significant difference.

## 5. Conclusions

In summary, we obtained a novel urease inhibitor of ruminal microbiota using an identified ruminal microbial metagenomic urease gene in a molecular docking based virtual screening. Compound 3-[1-[(aminocarbonyl)amino]-5-(4-methoxyphenyl)-1H-pyrrol-2-yl] propanoic acid (ChemDiv_ID: 6238-0047, IC_50_ = 65.86 μM) was found to be the most effective urease inhibitor, and it was able to lower the amount of urea degradation and ammonia production in rumen microbiota fermentation. The compound showed no cytotoxic effect on the gut epithelial Caco-2 cells. Molecular docking revealed that compound 6238-0047 could bind to both the active site cavity and the flap region of the urease active site. The strategy of using the gene information of the targeted bacteria to devise a molecular docking-based virtual screening of a particular chemical database to find compounds that could regulate a specific function of microbiota presented in this paper might also be implemented elsewhere. Compound 6238-0047 could serve as a lead compound for further modification to improve its urease inhibitory activity. Further animal assay needs to be conducted to verify the effectiveness and safety of this novel urease inhibitor.

## Figures and Tables

**Table 1:** Docking score and chemical formula of top 10 candidate compounds.

Rank	ChemDiv ID	Total Score	Structure
1	6238_0047	10.0992	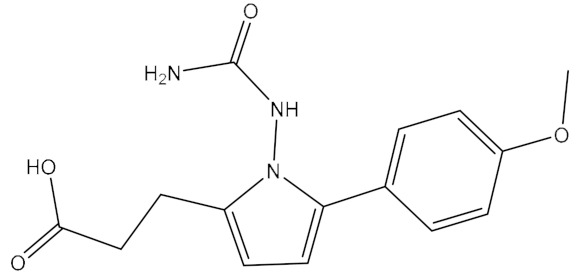
2	G279_1187	9.7817	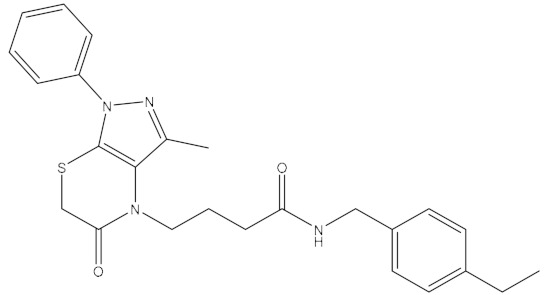
3	G819_0203	9.7376	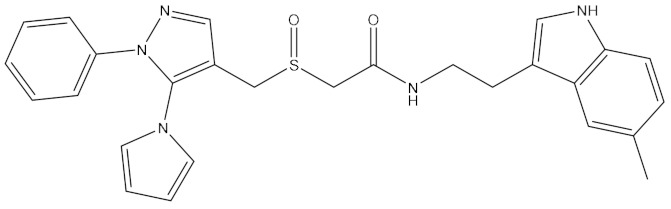
4	E734_1898	9.703	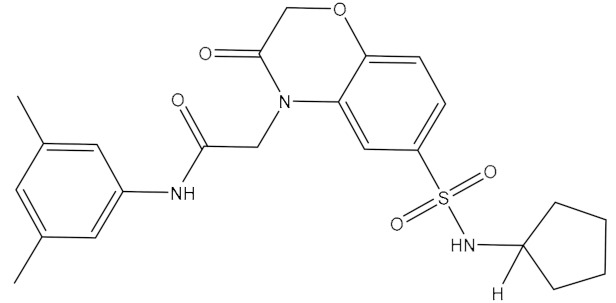
5	F046_0043	9.2562	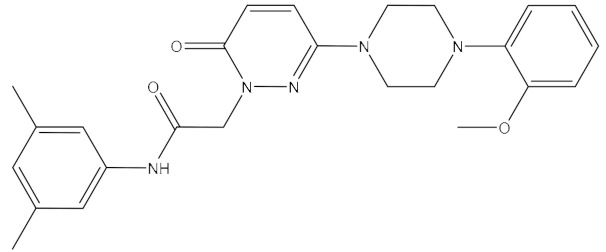
6	E551_0137	9.2236	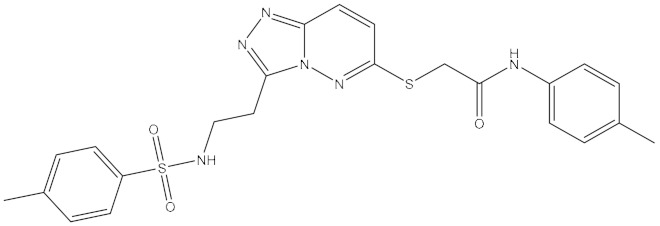
7	D146_0198	9.198	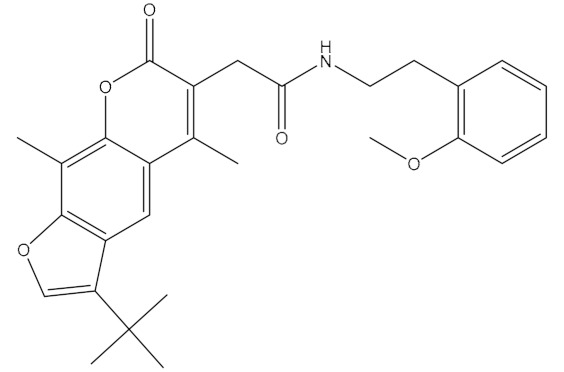
8	D481_1476	9.0675	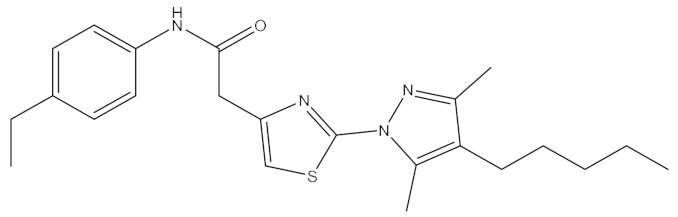
9	S576_0588	9.0028	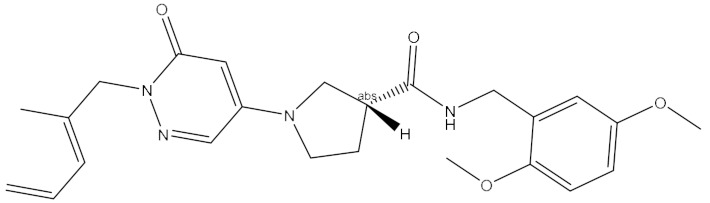
10	F046_0013	8.9094	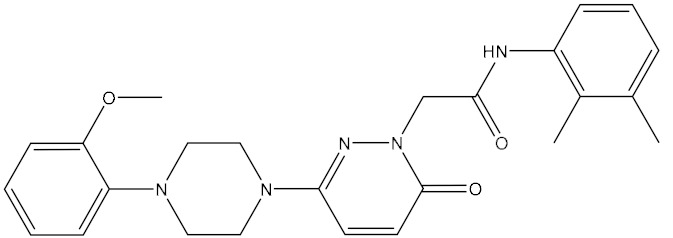

## Supplementary Materials

The following are available online at https://www.mdpi.com/1422-0067/21/17/6006/s1, Figure S1. The active site of ruminal metagenomic urease homology model. Figure S2. Binding mode of compounds 6238-0047 with the active site of ruminal metagenomic urease homology model. Table S1. Docking score and chemical formula of candidate compounds (top11-20).
