# Supplementary material for: A Novel Urease Inhibitor of Ruminal Microbiota Screened through Molecular Docking
Source: Int J Mol Sci. 2020 Aug 20;21(17):6006. doi: 10.3390/ijms21176006 (PMC7503308; doi:10.3390/ijms21176006)

Supplemental material

Table 1. Docking score and chemical formula of candidate compounds (top11-20).

Rank	ChemDiv ID	Total Score	Structure
11	E734_1954	8.8859	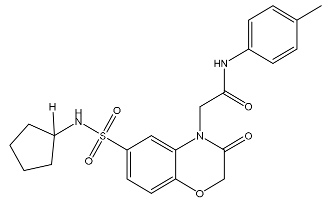
12	F744_0156	8.8662	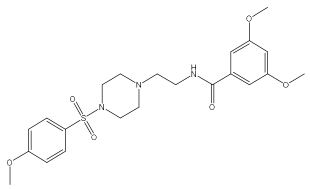
13	D475_1699	8.8557	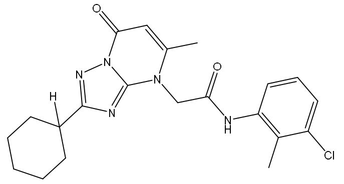
14	L879_0020	8.8402	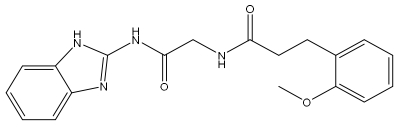
15	S576_0333	8.8174	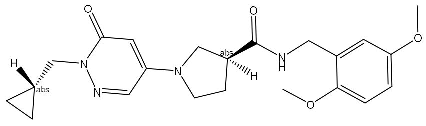
16	E699_2409	8.8162	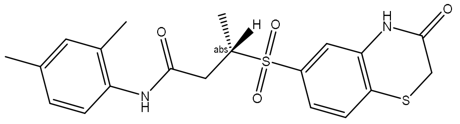

Rank	ChemDiv ID	Total Score	Structure
17	S576_0590	8.8084	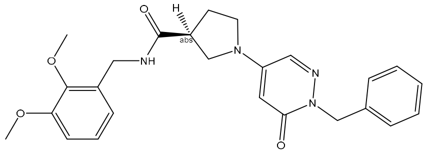
18	F046_0010	8.7929	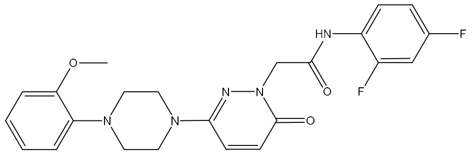
19	F711_0534	8.7106	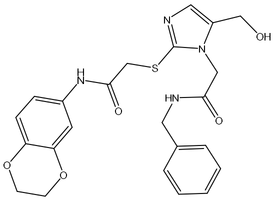
20	G406_2430	8.7087	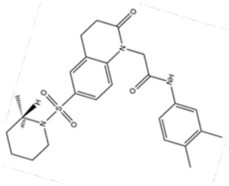

Figure S1. The active site of ruminal metagenomic urease homology model.

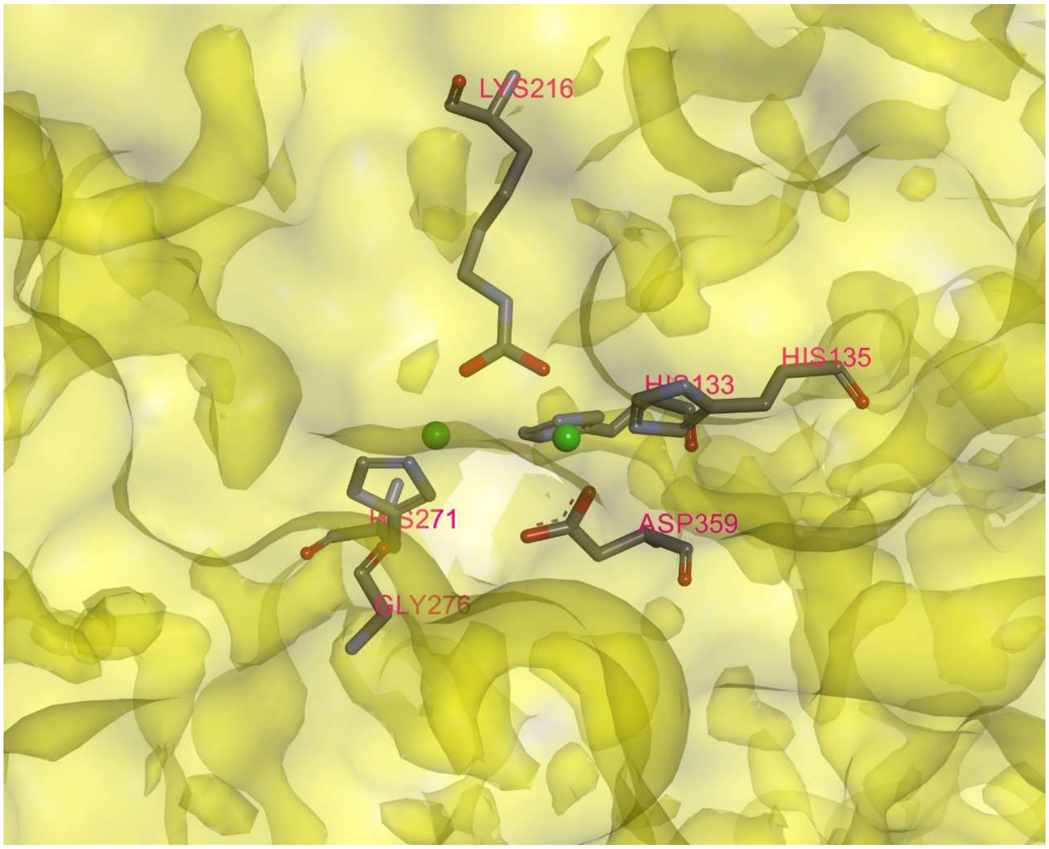

The active site of ruminal metagenomic urease homology model consists of two nickel ions (green spheres), bridged by Lys216, with one nickel bounds to His 133 and another nickel bonds to His 245. His 271 and Asp 359 also coordinates to one of the nickel ions.

Figure S2. Binding mode of compounds 6238-0047 with the active site of ruminal metagenomic urease homology model.

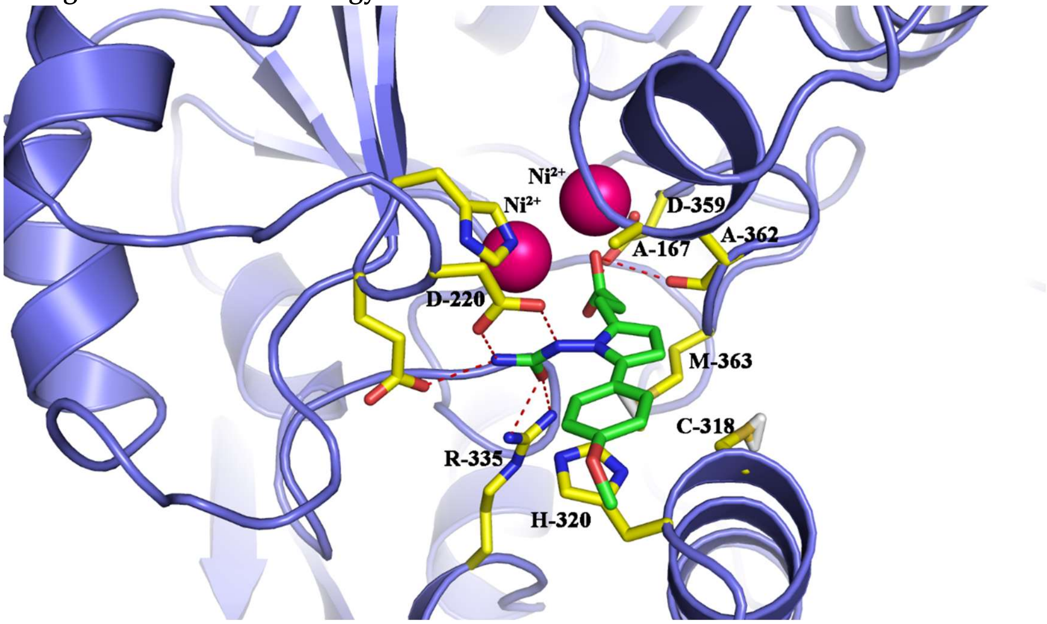

Binding mode of compounds 6238-0047 with the active site of ruminal metagenomic urease homology model was revealed by molecular docking. The active site is colored in light purple color while its critical amino acid residues are represented by yellow stick. The compound 6238-0047 is depicted in green color. while their hydrogen-bond-related functional groups are painted in red and blue respectively. The red dotted lines indicate where the hydrogen bonds formed. Two nickel atoms are demonstrated in rose color.

Detailed parameters for virtual screening

Virtual screening is usually realized by two methods:

a. Ligand-based methods, which rely on the similarity between the compounds of interest and the known active compounds. This method often resort to a technology named Pharmacophore.
b. Receptor-based methods, which rely on the complementarity of the compounds of interest with the binding site of the target protein. This methods usually based on a technology named Molecular docking.
In our study, we employed molecular docking technology to do virtual screening against ChemDiv compound database. This means that parameters and procedures for virtual screening are also for the molecular docking.
1. Parameters for compound filtering to obtain compounds with drug-like property.
①molecular weight <700
②ClogP -4 ~ 6
③Number of H-bond donors <6
④Number of H bond acceptors <15
⑤Number of rotatable bonds <10

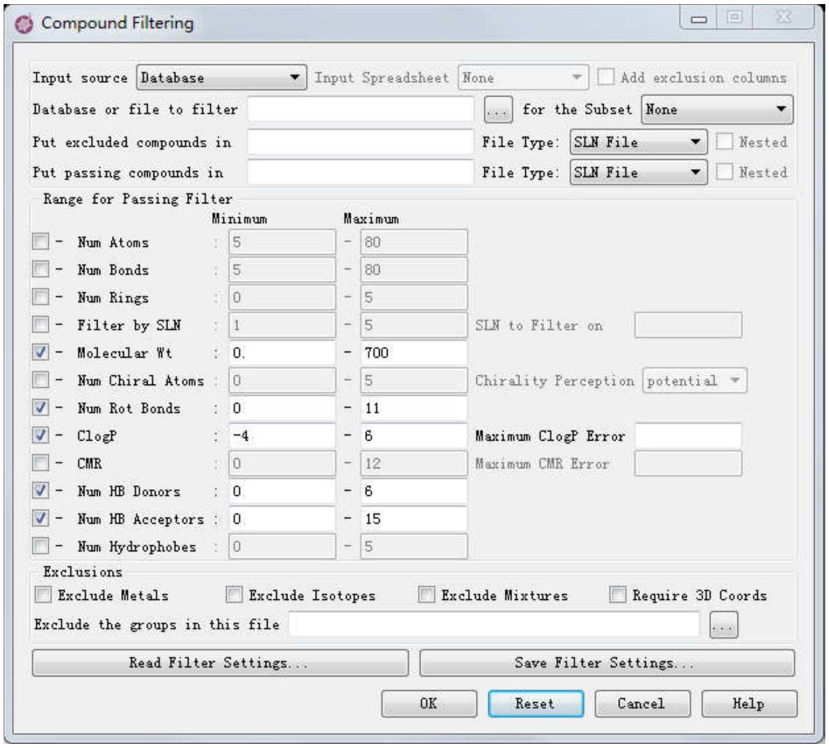

2. Parameters for the first round of virtual screening
The parameters used for the selection of 8753 compounds out of 0.87 million in the first round of virtual screening were listed as follows:
①"per-dock minimization" and "post-dock minimization" were selected.
②"max conformations per fragment" was set to 20.
③"max number of rotatable bonds per molecule" was set to 100.
④"maximum number of poses per ligand" was set to 10.

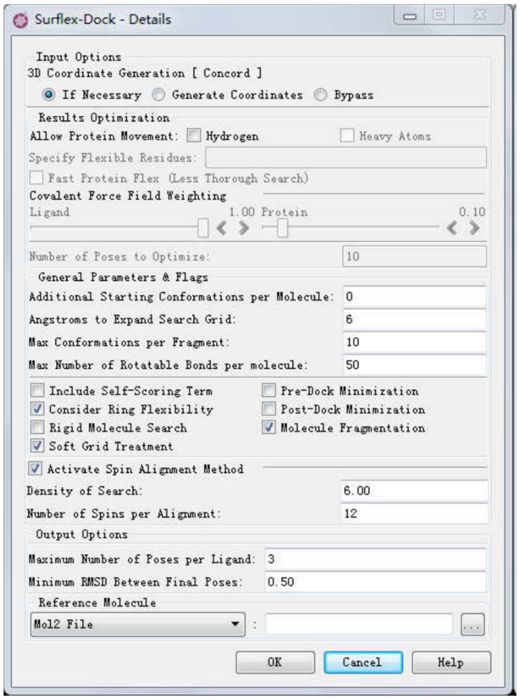

3. Parameters for the second round of virtual screening

The parameters used for the selection of 20 candidate compounds out of 8753 compounds in the second round of virtual screening were listed as follows:

①“per-dock minimization” and “post-dock minimization” were selected.

②“max conformations per fragment” was set to 20.

③“max number of rotatable bonds per molecule” was set to 100.

④“maximum number of poses per ligand” was set to 10.

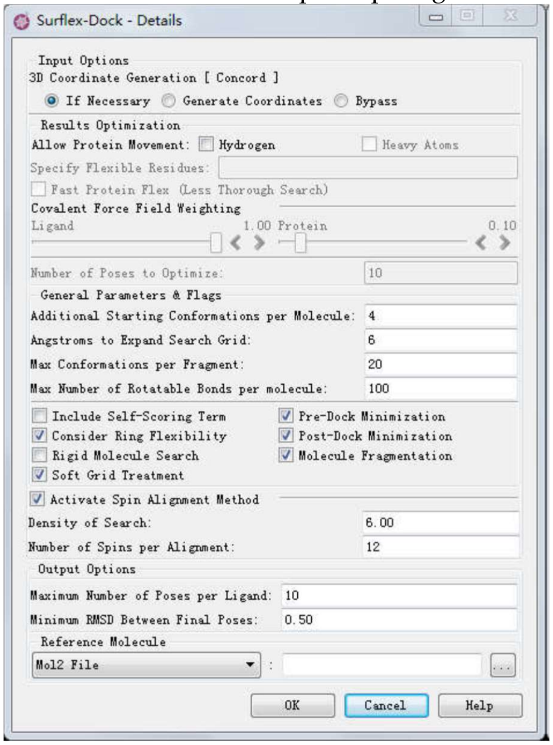

Supplement: Supplementary file 1 [file ijms-21-06006-s001.pdf]
